# The Time of Infection

**Published:** 1897-10

**Authors:** J. M. Selfridge


### "THE TIME OF INFECTION."

Dr. Selfridge, in discussing this subject, said:

"The therapeutics of Hahnemann challenge the world for their scientific exactness and the simplicity of their application.

"In his directions for the examination of patients, and for the proving of drugs, as well as for their application to the cure of disease, he stands to-day without a peer.

"When Hahnemann in *The Organon* and in his *Chronic Diseases* makes the statement that all chronic diseases owe their origin to three chronic miasms, viz., sycosis, syphilis, and psora, I accept the statement because it is possible for every careful observer to verify these deductions, which are the result of his vast experience and wonderful powers of observation.

"But when he makes the statement that both acute and chronic miasms are received into the system in '*one single moment*,' and that it enters the system through the nerves, I cannot accept his statement.

"He illustrates this thought by vaccination, but his admission that all these infectious diseases have a period of incubation is a source of weakness rather than strength to his argument. If miasmatic contact 'irrevocably, dynamically, communicates the disease to the vital force (to the whole nervous system) in the same moment,' why should there be any need of a period of incubation? 'The morbid fluid' is without doubt a disturbing element, but if communicated 'to the whole nervous system' in a single moment, the person affected ought to be instantaneously sick, whereas the converse is true.

"While the functions of the nervous system are to receive impressions and have a general supervision of vital dynamics, it is not one of its functions to carry 'morbid fluid' through the organism. This is clearly the office of the absorbents.

"Since the time of Hahnemann science has made discoveries, which, in some instances, elucidate his statements much better than he does himself. Since his time it has been discovered that there are such beings as microscopical germs. Now, while I do not believe that germs *per se* are the cause of diseases, I *do* believe they are *carriers* of poison, and that these ptomaines are disturbers of the vital processes.

"Take vaccination, the illustration named by Hahnemann. It has been demonstrated over and over again that 'cow-pox virus' will not produce the 'vaccine disease' unless germs are present in the 'morbid fluid,' and when introduced into the 'bloody scratch,' they carry with them these vaccine ptomaine or alkaloid poisons, which, in the process of incubation at the point of local contact, are set free, and, being taken up by the absorbents, are carried to the protoplasm in the primal cells, where all activities commence; but, instead of being converted by the protoplasm (which is the great store-house of vital energy) into bioplasm or living matter, they, being poisonous, disturb the movements of the molecules, and, of course, of the vital force, with which they are intimately connected, and the result is the whole organism becomes sick, *pari passu* with the local inflammation in vaccination, and of the chancre in syphilis.

"If contagion be taken into the system by insufflation, as in cases of whooping-cough, measles, chicken-pox, and small-pox, the same thing pertains, for the reason that the only avenue through which contagion, or any other substance, can reach the protoplasm, is by way of the absorbents. That it is through the absorbents is well illustrated by a case of sepsis.

"A lady, who at that time lived in San Francisco, pricked the middle finger of her left hand on the thorn of a cactus that grew in her garden. A fly doubtless had deposited some septic ptomaines on that thorn not long before the accident. The finger soon became swollen and painful, and the swelling and redness extended to the digital portion of the hand. The patient at the same time became chilly and feverish. About this time the late Dr. Pease was called in, and, after lancing the finger, gave the remedy that seemed to be indicated, but it did not stop the upward march of the septic poison, for the whole hand became involved, then the wrist, and the lower portion of the forearm. Inch by inch the redness and swelling had traveled from the finger upward. The progress was so rapid that in twelve hours the redness and swelling had extended from the wrist to the elbow. At this time I was called in consultation. The hand was spongy and of a purplish color. The arm was red and swollen to the elbow, where there was a distinct line of demarcation. The patient had a high fever, and was in a semi-comatose condition, with muttering delirium. The case was alarming. Something had to be done to stay the progress of the septic poison, which was traveling at the rate of an inch an hour.

"My suggestion was the application of a fly blister two inches wide, to be applied around the arm about four inches above the elbow. Under similar circumstances I had used the blister many times before with perfect success. I also advised scarifying the back of the hand to let out the pus and decomposed blood, which proved to be present in large quantity. The blister 'drew well,' and the redness and swelling continued up *to* the blister, but not *beyond* it. The indicated remedy was continued, and the patient recovered, but two weeks later the finger with its phalanx had to be amputated because of necrosis of the bone.

"You may ask, Do you consider the application of a blister under such circumstances good homœopathic practice? My answer is, It was good surgery, because the artificial inflammation caused by the blister closed the absorbents with inflammatory lymph and stopped the progress of the septic poison, which, but for that, would, undoubtedly, have proved fatal to the patient."

Dr. Martin asked—Then you do not agree with Hahnemann's theory?

Dr. Selfridge—I do not believe the whole system is infected at once. Inoculation takes place at once, but not infection.

Dr. Martin—Is not infection more a matter of degree? We are infected instantaneously, but the disease does not develop immediately. Tetanus infects instantly, but other poisons may take time to incubate.

Dr. Selfridge—Tetanus is not a poison! In poisons or disease ptomaines form and are carried by the lymphatics.

Dr. Martin—I do not care what system carries it! In the case of the Gila monster bite, the system is infected at once, but syphilis develops in thirty to forty days. Therefore, I consider it more a matter of degree.

Dr. Selfridge—These are not parallel cases. Rapid poisons are carried in a few seconds, but disease is not a parallel case.

Dr. Hohngren—In case of vaccination, if the portion inoculated be immediately taken away, the member be cut off, would a person develop any symptoms, or would he be proof against small-pox?

Dr. Manning—The trouble is in limiting the time of infection and time of incubation. I believe with Dr. Selfridge in instantaneous inoculation, but a period of time for the poison to reach the "whole" system. The time of infection then varies in length, but is this not rather a period of inoculation? Inoculation is instantaneous.

Dr. Augur—In cases of poison we know excision or cauterization destroys the poison, and if resorted to at once the system is not infected.

Dr. C. M. Selfridge—In cases of snake bite the system is infected at once.

Dr. J. M. Selfridge—No period of incubation exists in remedies, but even then I consider they are taken and conveyed by the absorptives. In vaccination the virus is taken up by lymphatics.

Dr. Augur—In all my cases of syphilis, all showed a want of cleanliness, from carelessness, intoxication, or absence of the necessary conditions. If aseptic measures were used at once would these cases develop?

Dr. Martin cited a case of personal injury during a *post-mortem*, in which septic symptoms rapidly developed, and he did not doubt but that he was infected all over, and at once.

Dr. Selfridge—I have had cases similar where I have applied pure Carbolic-acid to the wound two or three times, and no further symptoms developed, because it had not affected the whole system. It is possible to filter out germs and still have ptomaines. I believe in germs, but I believe in them more as a product of disease. They do carry disease, deposit it, and then it is carried through the system by absorptives.

This closed the discussion.

The meeting then adjourned till Friday, August 6th.

After the business part of the meeting we were surprised by a pleasant little feast in Dr. Martin's offices. After a pleasant hour spent over the feast and in conversation, Dr. Geo. H. Martin, acting as toast-master, gave each member a chance to distinguish himself or herself by speaking extemporaneously on subjects scientific and happily chosen. No one shirked the responsibility, except the toast-master, who lost his subject in the "shuffle" and in his modesty overlooked himself. He was finally called to time by the President, who demanded from him "Homœopathy in Nervous Diseases." Mention must also be made of an admirable poem by Dr. J. M. Selfridge on "Not Enough Homœopathy Taught."*

GUY E. MANNING,
*Secretary.*

---

FRIDAY, August 6th, 1897.

The regular semi-monthly meeting of *The Organon* and Materia Medica Club convened at the office of the Drs. Selfridge, Oakland. Drs. J. M. Selfridge, Augur, Hohngren, G. H. and E. F. Martin were present.

The Secretary being absent, Dr. G. H. Martin acted as Secretary *pro tem.*

---

*This poem will be found in the September number of THE HOMŒOPATHIC PHYSICIAN, page 369.

The minutes of the annual meeting, July 2d, read and approved.

Dr. Augur read a paper on "Sycosis and Syphilis," epitomizing and presenting clearly Hahnemann's ideas on the same, as expressed in his works.

In the discussion, other members gave their understanding of Hahnemann's meaning on certain points, but these did not differ materially, and all agreed that science had not changed, nor time altered greatly Hahnemann's doctrines on these two "chronic miasms."

Adjourned on motion till Friday, August 20th, 1897.

G. H. MARTIN,
*Secretary pro tem.*

